# Networked Pathological Mechanisms of Central Sympathetic Nervous System Regulation in Heart Failure and Novel Paradigms for Targeted Intervention

**DOI:** 10.3390/ijms27093924

**Published:** 2026-04-28

**Authors:** Zhengwei Li, Yi Yang, Renjun Wang

**Affiliations:** Department of Biotechnology, School of Life Science, Jilin Normal University, Siping 136000, China; lizhengwei@mails.jlnu.edu.cn

**Keywords:** heart failure, central sympathetic nervous regulation, brain-heart axis, glial cell activation, endoplasmic reticulum stress, extracellular vesicles

## Abstract

Excessive activation of the sympathetic nervous system is a prominent contributor linked to heart failure (HF) progression. Pathological remodeling of the central nervous system represents a plausible upstream event associated with central sympathetic hyperactivity, whereas dysfunction of the brain–heart axis may act as a pivotal hub involved in this pathological process. This review systematically summarizes the functional characteristics of major sympathetic regulatory nuclei under HF, including the subfornical organ (SFO), paraventricular nucleus of the hypothalamus (PVN), rostral ventrolateral medulla (RVLM), and nucleus tractus solitarius (NTS). Following the pathological logic from upstream initiation to inter-organ closed-loop responses, seven interconnected pathological mechanisms are analyzed: glial cell activation and neuroinflammation, endoplasmic reticulum stress, renin–angiotensin system (RAS) imbalance, abnormal signaling pathways and transcription factors, impaired neuronal microenvironment homeostasis, dysregulated post-transcriptional and post-translational modifications, and extracellular vesicle-mediated inter-organ signal transmission. Their cross-regulation and positive feedback amplification effects are highlighted. Multidimensional central-targeted intervention strategies established on this basis possess important fundamental significance and translational potential. This review also discusses current scientific challenges and prospects for interdisciplinary frontiers, providing theoretical references and practical insights for central regulation research in HF and its precise clinical translation.

## 1. Introduction

Heart failure (HF) represents the end-stage outcome of various cardiovascular diseases, characterized primarily by impaired myocardial systolic and diastolic function, neurohumoral disorders, and insufficient peripheral circulatory perfusion [1]. Its global prevalence continues to rise, accompanied by persistently high mortality and readmission rates, rendering HF a major challenge urgently requiring resolution in contemporary cardiovascular research [2,3,4]. Autonomic imbalance constitutes a central and persistent neurohumoral abnormality in the pathogenesis of HF, mainly manifested as pathological upregulation of the sympathetic nervous system (SNS) and parasympathetic inhibition, among which chronic overactivation of the SNS is strongly associated with and may contribute to disease progression [5,6,7]. In the early stage of the disease, this imbalance transiently compensates hemodynamics by increasing heart rate and enhancing myocardial contractility. However, prolonged overactivation induces myocardial remodeling and cardiac electrophysiological disorders, and acts synergistically with activation of the renin–angiotensin–aldosterone system (RAAS). Through the vicious cycle of “sympathetic excitation–myocardial injury–further sympathetic hyperactivity”, it ultimately promotes HF deterioration, triggers severe arrhythmias, and even leads to sudden cardiac death [6].

Previous studies have mostly focused on peripheral targets such as peripheral sympathetic nerve terminals [8,9,10,11,12,13] and myocardial adrenergic receptors [14,15,16,17,18,19]. Although some peripheral regulatory mechanisms of sympathetic activation have been elucidated, they cannot fully explain the persistent, systemic, and centrally derived characteristics of sympathetic drive in HF [20]. Recent studies have confirmed that the central nervous system (CNS) serves as the core regulatory and integrative center of sympathetic nerve activity [21]. Key nuclei including the brainstem and hypothalamus can precisely regulate sympathetic efferent impulses through complex neural circuits and molecular signaling pathways [22]. Under HF conditions, structural remodeling and functional abnormalities occur in the CNS, which are closely linked to and may participate in initiating and sustaining excessive sympathetic activation [23]. Regarding the central regulatory mechanisms of sympathetic activation, current studies have established the crucial roles of core dimensions including neuroinflammation, glial cell activation, and imbalance of central signaling networks. The molecular and cellular mechanisms underlying the brain–heart axis regulation have been continuously elucidated, driving the field from basic mechanistic exploration to translational research and providing novel targets and strategies for precise neuromodulatory therapy of HF [23].

At present, numerous key scientific questions regarding the central regulation of sympathetic activation in HF remain to be clarified. The precise connectivity and functional segregation of central sympathetic regulatory circuits have not been fully defined; there is no unified consensus on the crosstalk and hierarchical effects among multiple regulatory mechanisms, and central-targeted intervention strategies still face bottlenecks including blood–brain barrier penetration, insufficient target specificity, and poor long-term safety. Accordingly, this review systematically summarizes the physiological functions of core nuclei involved in CNS regulation of sympathetic nerves, dissects the key pathological mechanisms underlying central sympathetic activation in HF layer by layer, summarizes the therapeutic strategies and clinical translational progress of central-targeted interventions, and prospects the research challenges and frontier directions in this field. It aims to provide theoretical references and insights for further understanding the central nature of sympathetic dysregulation in HF and developing precise and efficient novel neuromodulatory therapies.

## 2. Core Nuclei and Neural Circuit Architecture of Central Sympathetic Regulation

**Figure 1:**
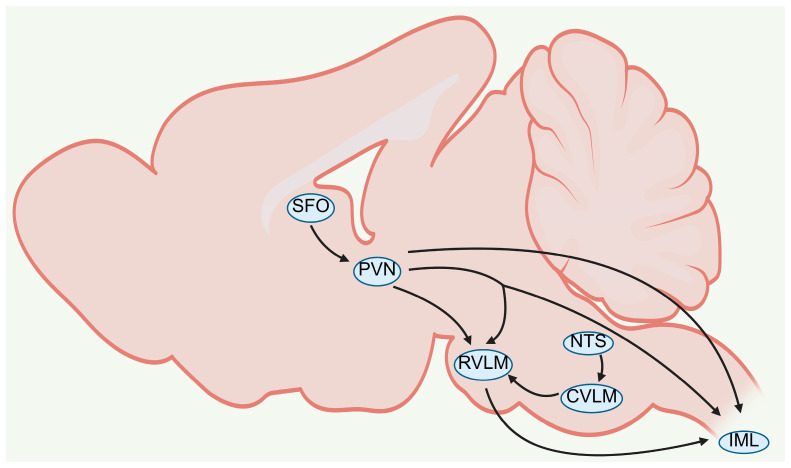
Core brain nuclei and neural circuits regulating sympathetic nerve activity in the central nervous system. Schematic depicting the core network and projections controlling sympathetic outflow. Note: arrows indicate directional neural projections and signal transmission between the listed brain nuclei. Abbreviations: SFO (subfornical organ); PVN (paraventricular nucleus of the hypothalamus); RVLM (rostral ventrolateral medulla); NTS (nucleus tractus solitarius); CVLM (caudal ventrolateral medulla); IML (intermediolateral column of the spinal cord). Created in BioRender. Zhengwei Li, Renjun Wang. (2026) https://BioRender.com/6j7dk3s (accessed on 25 March 2026).

The paraventricular nucleus of the hypothalamus (PVN), rostral ventrolateral medulla (RVLM), and nucleus tractus solitarius (NTS) serve as the core integrative and regulatory nuclei for CNS modulation of sympathetic nerve activity (SNA) [24]. The subfornical organ (SFO) acts as a critical sensory interface for peripheral signal transmission into the CNS [25,26], whereas the intermediolateral column of the spinal cord (IML) constitutes the final hub for sympathetic signal transmission to peripheral target organs [27]. These nuclei are connected via precise neural circuits, forming a complete network for central sympathetic regulation (Figure 1).

Located around the third ventricle and characterized by the absence of the blood–brain barrier and high vascularization, the SFO directly senses changes in the peripheral environment such as extracellular sodium concentration, and undertakes signal sensing and transmission between the blood and the CNS [25,26]. Its afferent signals positively regulate the neuronal activity of the downstream PVN [28]. The PVN is a key integrative nucleus in central sympathetic regulation, critically involved in SNA control and extracellular fluid volume homeostasis [25,29]. It constructs a complex regulatory network through diverse neuronal subtypes and fiber projections: neurons projecting to the spinal cord include rapidly conducting myelinated and slowly conducting unmyelinated subtypes, both of which exhibit spontaneous firing correlated with renal sympathetic nerve activity and mediate sympathetic vasomotor regulation and non-vasomotor sympathetic modulation, respectively [30]. Neurons projecting to the RVLM are classified into PVN-RVLM single-projecting and PVN-RVLM/IML branching-projecting subtypes, which display similar fiber properties and firing frequencies, with most spontaneous firing associated with renal sympathetic nerve activity, jointly contributing to baseline SNA maintenance and baroreflex modulation [31]. Furthermore, the PVN establishes direct nerve fiber connections with the NTS and IML, serving as a central integrative node within the central sympathetic regulatory network [25,29].

As the core receiving nucleus for sympathetic afferent signals, the NTS directly accepts input from cardiopulmonary afferent nerves and the area postrema (AP), and indirectly modulates the sympathetic regulatory function of the RVLM by regulating neuronal activity in the caudal ventrolateral medulla (CVLM) [32,33]. The RVLM, known as the “sympathetic center”, plays a decisive role in tonic maintenance and reflex regulation of SNA [27]. It receives afferent signals from the PVN and forms functional connections with the IML; meanwhile, the IML is also directly innervated by the PVN [34,35]. Ultimately, the IML integrates signals from hierarchical sympathetic regulatory nuclei including the PVN and RVLM, activates postganglionic sympathetic neurons innervating peripheral target organs, and completes the overall central regulation and output of sympathetic nerve activity.

## 3. Networked Pathological Mechanisms of Central Sympathetic Overactivation in Heart Failure (HF)

The pathological mechanisms underlying central sympathetic overactivation in heart failure are sequentially initiated, amplified, and executed rather than functioning in parallel. To establish a clear hierarchical framework and reflect a systematic perspective, these mechanisms are organized into three functional stages: initiation stage, amplification stage, and execution stage. Crosstalk, synergistic regulation, and vicious cycle effects among these stages cooperatively drive the persistent progression of central sympathetic hyperactivity, forming a networked pathological regulatory system.

### 3.1. Initiation Stage: Peripheral Signal Sensing and Central Initial Activation

#### 3.1.1. Extracellular Vesicle-Mediated Trans-Organ Signaling: A Critical Bridge in the Pathological Vicious Cycle of the Brain-Heart Axis

**Figure 2:**
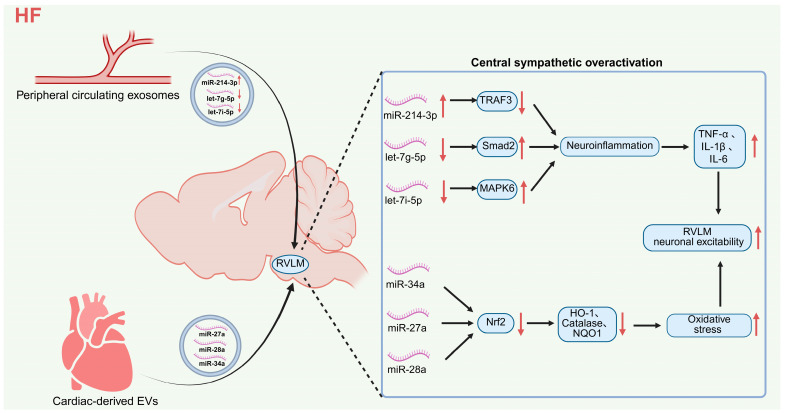
Mechanisms by which extracellular vesicles mediate oxidative stress and neuroinflammation in the RVLM to synergistically drive central sympathetic overactivation in heart failure. Schematic illustrating EV-induced molecular disturbances promoting sympathetic overactivation. Note: ↑ indicates upregulation, increased activity or enhanced effect; ↓ indicates downregulation, impaired function or weakened inhibitory effect; arrows indicate the direction of signal transmission, regulation or action. Abbreviations: HF (heart failure); EVs (extracellular vesicles); RVLM (rostral ventrolateral medulla); miR (microRNA); Nrf2 (nuclear factor erythroid 2-related factor 2); HO-1 (heme oxygenase-1); NQO1 (NAD(P)H quinone dehydrogenase 1); TRAF3 (tumor necrosis factor receptor-associated factor 3); Smad2 (SMAD family member 2); MAPK6 (mitogen-activated protein kinase 6); TNF-α (tumor necrosis factor-α); IL-1β (interleukin-1β); IL-6 (interleukin-6). Created in BioRender. Zhengwei Li, Renjun Wang. (2026) https://BioRender.com/osxljiw (accessed on 25 March 2026).

Extracellular vesicles (EVs, including exosomes) serve as core functional mediators of central-peripheral trans-organ signaling in heart failure, and act as a critical bridge linking peripheral cardiac pathological injury to central sympathetic overactivation. EVs can selectively carry functional miRNAs across the blood–brain barrier, target key nuclei involved in central sympathetic regulation, and mediate sympathetic hyperactivity via oxidative stress and neuroinflammatory pathways (Figure 2). This mechanism has been validated in clinical patients with ischemic heart failure and exhibits clear potential for clinical translation [36,37].

In the oxidative stress pathway, heart-derived EVs from CHF rats selectively enrich miRNAs targeting nuclear factor erythroid 2-related factor 2 (Nrf2), including miR-27a, miR-28a, and miR-34a [38]. After being released into the circulation, these EVs can cross the blood–brain barrier and be taken up by sympathetic premotor neurons in the RVLM. The enclosed miRNAs inhibit Nrf2 translation, resulting in downregulated expression of downstream antioxidant proteins including heme oxygenase-1 (HO-1), catalase, and NAD(P)H quinone dehydrogenase 1 (NQO1). This further causes oxidative stress imbalance and aggravated DNA/RNA oxidative damage in the RVLM, ultimately mediating central sympathetic excitation by increasing RSNA and elevating plasma norepinephrine [38,39].

Meanwhile, the miRNA expression profile in circulating exosomes from CHF rats also exhibits distinct alterations, characterized by significantly upregulated miR-214-3p and markedly downregulated let-7g-5p and let-7i-5p [40]. These exosomes can likewise cross the blood–brain barrier and target the RVLM. MiR-214-3p enhances central inflammatory responses by targeting and inhibiting TNF receptor-associated factor 3 (Traf3). In contrast, the reduced expression of let-7g-5p and let-7i-5p is predicted to weaken their anti-inflammatory effects on mothers against decapentaplegic homolog 2 (Smad2) and mitogen-activated protein kinase 6 (Mapk6), respectively. Based on target validation and in vitro evidence from [40], we propose that these miRNA changes may contribute to excessive inflammation in the RVLM, accompanied by significantly elevated levels of TNF-α, interleukin-1β (IL-1β), and interleukin-6 (IL-6). This, in turn, may increase neuronal excitability and further promote sympathetic hyperactivity [40].

However, the in vivo transcytosis efficiency of EVs across the blood–brain barrier and their quantitative delivery efficiency to RVLM neurons under heart failure remain unclear [38]. Under physiological conditions, circulating EVs are rapidly cleared by peripheral organs such as the liver and spleen [41], whereas direct experimental evidence regarding the in vivo clearance and distribution characteristics of EVs during heart failure is lacking. Recent studies have confirmed that acute systemic inflammation significantly inhibits the hepatic clearance rate of circulating EVs, prolongs their plasma half-life by hundreds of times, and markedly increases EV accumulation in extrahepatic organs including the brain and lungs [42], suggesting that the chronic inflammatory state associated with heart failure may similarly alter the in vivo clearance and tissue distribution patterns of EVs. Compared with peripheral clearance organs such as the liver and spleen, accurate in vivo quantitative data on the proportion of circulating EVs that actually reach the RVLM parenchyma are unavailable. Meanwhile, heart failure can increase blood–brain barrier permeability [43,44], which may not only facilitate the entry of EVs into the brain but also allow free miRNAs in plasma to penetrate the compromised barrier into the central nervous system. The relative contributions of EV-encapsulated miRNAs and free circulating miRNAs to central sympathetic overactivation during heart failure warrant further investigation.

#### 3.1.2. Central Glial Cell Activation and Neuroinflammatory Cascade: A Potential Upstream Contributor to Sympathetic Overactivation

**Figure 3:**
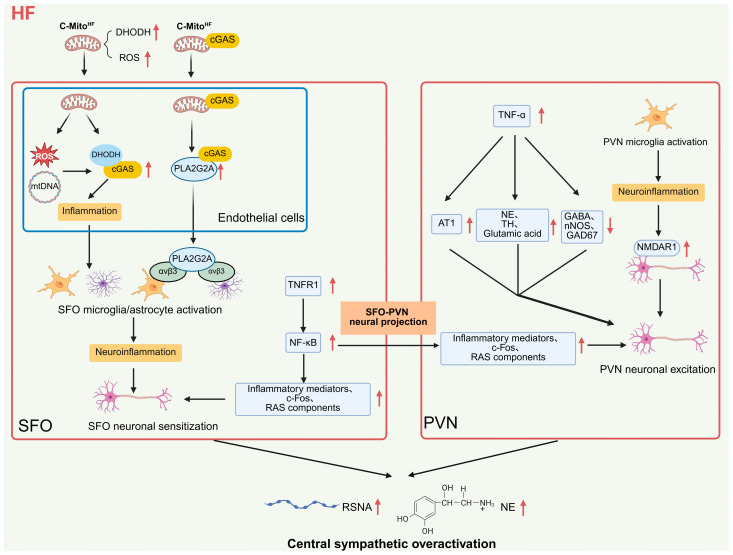
Pathological mechanisms of SFO-PVN axis neuroinflammation mediating central sympathetic overactivation in heart failure. Schematic illustrating the inflammatory cascade driving sympathetic hyperactivity. Note: ↑ indicates upregulation, increased activity or enhanced effect; ↓ indicates downregulation or impaired function; arrows indicate the direction of signal transmission, activation or regulation. Abbreviations: HF (heart failure); C-MitoHF (heart failure-associated damaged circulating mitochondria); DHODH (dihydroorotate dehydrogenase); ROS (reactive oxygen species); cGAS (cyclic GMP-AMP synthase); mtDNA (mitochondrial DNA); PLA2G2A (secretory phospholipase A2 group IIA); SFO (subfornical organ); PVN (paraventricular nucleus of the hypothalamus); TNF-α (tumor necrosis factor-α); AT1 (angiotensin II type 1 receptor); NE (norepinephrine); TH (tyrosine hydroxylase); GABA (γ-aminobutyric acid); nNOS (neuronal nitric oxide synthase); GAD67 (67 kDa glutamate decarboxylase); NMDAR1 (N-methyl-D-aspartate receptor 1); TNFR1 (tumor necrosis factor receptor 1); NF-κB (nuclear factor-κB); RAS (renin-angiotensin system); RSNA (renal sympathetic nerve activity). Created in BioRender. Zhengwei Li, Renjun Wang. (2026) https://BioRender.com/s0e6j30 (accessed on 25 March 2026).

Abnormal activation of central glial cells (microglia and astrocytes) and the subsequent central neuroinflammation are closely associated with upstream pathological events for central sympathetic overactivation in HF. This conclusion is a well-established consensus across multiple preclinical models. It can amplify sympathetic efferent effects by potentially enhancing the excitability of presympathetic neurons and regulating downstream signaling pathways, thereby promoting the progression of HF (Figure 3) [23,45].

Under HF conditions, injured circulating mitochondria (C-MitoHF) represent an important inducer of central glial cell activation and neuroinflammation initiation. They can specifically target endothelial cells in the SFO and trigger inflammatory cascades through two distinct molecular mechanisms. After C-MitoHF enters SFO endothelial cells in mice with HF, it not only increases reactive oxygen species (ROS) levels and promotes the release of cytoplasmic free double-stranded DNA (dsDNA), but also directly interacts with cyclic GMP-AMP synthase (cGAS) in endothelial cells via its highly expressed dihydroorotate dehydrogenase (DHODH). This interaction synergistically activates the cGAS proinflammatory signaling pathway, inducing endothelial inflammation [46]. This endothelial inflammation further activates microglia/astrocytes, triggering neuroinflammation in the SFO and sensitizing presympathetic neurons, ultimately mediating central sympathetic overactivation [46]. In rat models of HF, however, C-MitoHF interacts with secreted phospholipase A2 group IIA (PLA2G2A) in SFO endothelial cells via membrane-bound cGAS and promotes its expression and secretion. Secreted PLA2G2A then activates integrin αvβ3 on microglia/astrocytes to induce neuroinflammation, which similarly mediates central sympathetic overactivation by sensitizing presympathetic neurons [47]. However, these two pathways represent recently identified mechanisms that currently rely on limited preclinical evidence and await independent validation.

Abnormal expression and signaling of proinflammatory cytokines constitute a key step in amplifying neuroinflammation and mediating sympathetic activation following central glial cell activation in HF. This effect forms a ligand–receptor-mediated upstream–downstream regulatory network in the two core nuclei, SFO and PVN. High expression of tumor necrosis factor-α (TNF-α) in the PVN of HF rats upregulates angiotensin II type 1 receptor (AT1R) expression and modulates neurotransmitter balance within the nucleus, resulting in elevated glutamate, norepinephrine, and tyrosine hydroxylase (TH) as well as reduced γ-aminobutyric acid (GABA), neuronal nitric oxide synthase (nNOS), and 67 kDa glutamate decarboxylase (GAD67). These changes directly increase renal sympathetic nerve activity (RSNA), thereby mediating central sympathetic overactivation [48]. As the classical receptor for TNF-α, aberrant expression and activation of tumor necrosis factor receptor 1 (TNFR1) in the SFO of HF rats enhance nuclear factor-κB (NF-κB) activity in the PVN via SFO-PVN neural projections. This synchronously elevates inflammatory mediators, components of the renin-angiotensin system (RAS), and c-Fos protein expression in both nuclei, further amplifying the pathological effects beyond local TNF-α regulation in the PVN. Ultimately, it triggers sympathetic hyperactivity, elevated plasma norepinephrine, and aggravated cardiac dysfunction [49]. At present, all the above conclusions are derived from animal studies, and direct clinical evidence from human CNS tissues or cerebrospinal fluid remains lacking.

Neuroinflammation mediated by microglial activation in the PVN can directly induce overactivation of distinct sympathetic branches by regulating specific receptor expression and activating related signaling pathways, and is closely associated with the occurrence and development of arrhythmias. In rats with chronic HF, enhanced microglial inflammation in the PVN is accompanied by upregulated expression of N-methyl-D-aspartate receptor 1 (NMDAR1). Activation of NMDAR1 in the PVN directly promotes increased sympathetic nerve activity, and microglia-mediated inflammation further modulates NMDAR1 expression. Their synergistic effects elevate renal sympathetic nerve activity (RSNA) as well as norepinephrine levels in plasma and cerebrospinal fluid, ultimately mediating central sympathetic overactivation [50]. In addition, abnormal microglial activation and subsequent neuroinflammation in the PVN of HF rats also enhance sympathetic activity in the left stellate ganglion (LSG), inducing central sympathetic overactivation and thereby promoting the development of ventricular arrhythmias [51]. The causal relationship between neuroinflammation in the PVN and clinical arrhythmias remains to be confirmed in humans.

### 3.2. Amplification Stage: Pathological Cascade and Positive Feedback Amplification

#### 3.2.1. Central Endoplasmic Reticulum Stress (ERS): A Secondary Pathological Amplifier of Sympathetic Overactivation

**Figure 4:**
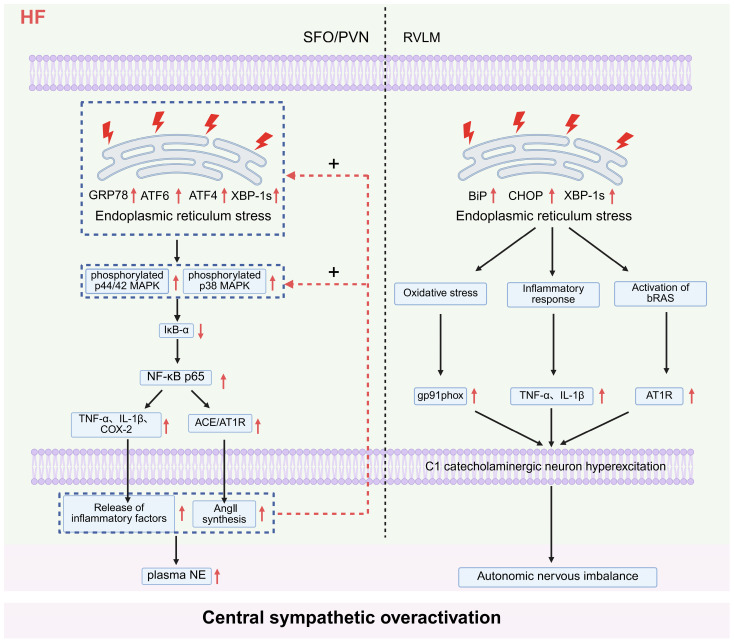
Pathological mechanisms by which endoplasmic reticulum stress in SFO/PVN and RVLM synergistically mediates central sympathetic overactivation in heart failure. Schematic showing ERS-driven pathological signaling in key brain nuclei. Note: ↑ indicates upregulation, increased activity or enhanced effect; ↓ indicates downregulation or impaired function; solid arrows indicate the direction of signal transmission, activation or regulation; red dashed arrows indicate positive feedback regulation (+). Abbreviations: HF (heart failure); SFO (subfornical organ); PVN (paraventricular nucleus of the hypothalamus); RVLM (rostral ventrolateral medulla); GRP78 (glucose-regulated protein 78); ATF6 (activating transcription factor 6); ATF4 (activating transcription factor 4); XBP-1s (spliced X-box binding protein 1); BiP (binding immunoglobulin protein); CHOP (C/EBP-homologous protein); p44/42 MAPK (p44/42 mitogen-activated protein kinase); p38 MAPK (p38 mitogen-activated protein kinase); IκB-α (nuclear factor-kappa B inhibitor alpha); NF-κB p65 (nuclear factor-kappa B p65 subunit); TNF-α (tumor necrosis factor-alpha); IL-1β (interleukin-1beta); COX-2 (cyclooxygenase-2); ACE (angiotensin-converting enzyme); AT1R (angiotensin II type 1 receptor); AngII (angiotensin II); NE (norepinephrine); gp91phox (nicotinamide adenine dinucleotide phosphate oxidase subunit gp91phox); bRAS (brain renin-angiotensin system). Created in BioRender. Zhengwei Li, Renjun Wang. (2026) https://BioRender.com/fc074xg (accessed on 25 March 2026).

Central ERS may contribute to the pathological processes involved in synergistic mediation of sympathetic overactivation by diverse cardiovascular regulatory nuclei in the brain during HF. Through nucleus-specific pathway regulation, it forms a cascade reaction of “initiation activation–pathway mediation–effect amplification”, thereby promoting the progression of HF (Figure 4). Current evidence suggests that ERS amplifies sympathetic hyperactivity via inflammatory and RAS pathways, whereas its specific regulatory patterns differ across distinct HF models.

In the SFO and PVN of rats with HF, ERS was significantly enhanced (upregulated expression of core markers GRP78, ATF6, ATF4, XBP-1s, etc.), which directly activated the p44/42 and p38 mitogen-activated protein kinase (MAPK) signaling pathways. This pathway activated NF-κB (marked by activation of the p65 subunit) by downregulating nuclear factor-κB inhibitor α (IκB-α). On one hand, it promoted the expression of TNF-α, IL-1β, and cyclooxygenase-2 (COX-2); on the other hand, it upregulated key components of the RAS including angiotensin-converting enzyme (ACE) and AT1R, ultimately increasing plasma norepinephrine (NE) levels and enhancing central sympathetic nerve activity [52]. Notably, AngII and inflammatory cytokines (TNF-α, IL-1β) synthesized in the SFO/PVN regions via the above pathways can act as potent inducers of ERS, which in turn further activate the p44/42 and p38 MAPK pathways. Meanwhile, by amplifying ERS effects, they sustainably upregulate the expression of markers such as GRP78 and ATF6, forming a positive feedback loop: “ERS activation initiates the MAPK pathway, which further induces inflammation and RAS activation, ultimately aggravating ERS in return”. This loop continuously amplifies pathological effects and promotes the progression of HF [53]. Of note, experimental evidence supporting the above pathological pathway is mainly derived from pharmacological inhibitor interventions and has not been verified by specific gene-targeting approaches; therefore, potential non-specific effects cannot be fully excluded.

Interestingly, the mechanism by which ERS mediates sympathetic activation is not limited to the SFO and PVN. Significant abnormal activation of ERS is also present in the RVLM of the brainstem in rats with high-output HF, as reflected by upregulated mRNA and protein levels of markers including BiP, CHOP, and XBP-1s. ERS in this region can induce abnormal excitability of C1 catecholaminergic neurons in the RVLM via a synergistic pathway involving oxidative stress (elevated gp91phax expression), inflammatory response (increased release of TNF-α and IL-1β), and activation of the brain RAS (upregulated AT1R), thereby enhancing central sympathetic nerve activity. This ultimately leads to autonomic imbalance and respiratory dysfunction, contributing to the pathological progression of HF [54]. Notably, the pathological effects of ERS in RVLM are mainly observed in high-output HF models, but not consistently validated in other models including ischemic HF, suggesting that its role may exhibit marked model dependence and nucleus specificity.

#### 3.2.2. Central Renin-Angiotensin System (RAS) Imbalance: A Key Neurohumoral Hub for Abnormal Sympathetic Regulation

Dysfunction of the central RAS may be implicated as a prominent neurohumoral mechanism in central sympathetic overactivation in HF. Its regulatory effects are primarily mediated by core cardiovascular regulatory nuclei including the PVN, SFO, RVLM, and NTS. Receptor expression imbalance, abnormal signaling pathways, and disrupted reflex regulation collectively contribute to the abnormal enhancement of SNA (Table 1).

PVN, as the core integrative nucleus for central RAS regulation of sympathetic activity, exhibits dysfunction that serves as an important initiating factor for sympathetic activation. In rats with chronic HF, elevated expression of angiotensin-(1-7) (Ang-(1-7)) in the PVN enhances cardiac sympathetic afferent reflex and amplifies effects synergistically with Ang II via activation of the Mas receptor through the cyclic adenosine monophosphate-protein kinase A (cAMP-PKA) pathway and nicotinamide adenine dinucleotide phosphate (NAD(P)H) oxidase-derived superoxide anion, increasing renal sympathetic nerve activity and mediating central sympathetic overactivation [55]. Meanwhile, downregulated expression of angiotensin-converting enzyme 2 (ACE2), Ang-(1-7) receptor, and neuronal nitric oxide synthase (nNOS) in the PVN impairs central nitric oxide (NO)-mediated sympathetic inhibitory pathways, further augmenting sympathetic excitation [56]. Furthermore, activation of NF-κB in the PVN of HF rats mediates crosstalk between RAS and proinflammatory cytokines (PICs), upregulates AT1R, and induces oxidative stress, in which ROS further activates NF-κB to form a vicious cycle, ultimately driving PVN-dependent neurohumoral excitation and central sympathetic activation [57].

As a key brain region for peripheral signal sensing, abnormal RAS in the SFO also contributes to sympathetic regulation. In the SFO of rats with chronic HF, expression of AT1R is significantly upregulated, and the tonic activation by endogenous Ang II is enhanced, leading to increased activation of SFO neurons, thereby elevating renal sympathetic nerve activity, blood pressure, and heart rate, resulting in SFO-dependent central sympathetic overactivation [58].

As the final executive center for sympathetic outflow, imbalanced RAS receptors in the RVLM directly exacerbates sympathetic excitation. In the RVLM of rats with chronic HF, AT1R is upregulated and angiotensin II type 2 receptor (AT2R) is downregulated, leading to an imbalance in their expression. Upregulated AT1R produces sympathoexcitatory effects, whereas downregulated AT2R eliminates its sympathoinhibitory effects mediated via the arachidonic acid metabolic pathway. These dual mechanisms collectively promote central sympathetic overactivation [59].

NTS participates in central RAS-mediated sympathetic activation through reflex regulatory pathways. In the NTS of rats with chronic HF, upregulated AT1R enhances the interaction between cardiac sympathetic afferent reflex (CSAR) and chemoreflex, amplifying sympathoexcitatory effects via an NTS-AT1R-dependent mechanism [60]. Other studies have confirmed that local RAS activation and upregulated angiotensin-converting enzyme (ACE) in the NTS of HF rats increase RSNA and arterial pressure through endogenous Ang II acting on AT1R [61]. These two pathways collectively mediate central sympathetic overactivation.

Collectively, most existing studies have focused on RAS abnormalities within individual nuclei, while the synergistic regulation and signal integration among multiple nuclei remain poorly systematically elucidated; further investigation is warranted to validate the clinical translational value of these findings.

#### 3.2.3. Abnormal Central Core Signaling Pathways and Transcription Factors: Intermediate and Amplifying Carriers of Pathological Signals

During HF, multiple signaling pathways and transcription factors in the PVN are abnormally activated and interactively regulated. They collectively mediate central sympathetic overexcitation by altering neurotransmitter balance, inducing neuroinflammation, and triggering oxidative stress (Table 2).

Within MAPK-related pathways, abnormal activation of the extracellular signal-regulated kinase 1/2 (ERK1/2) MAPK signaling pathway occurs in the PVN of rats with heart failure following myocardial infarction. This upregulates the expression of RAS components and inflammatory mediators, increases excitability of PVN neurons, elevates plasma norepinephrine levels, and thereby enhances sympathetic drive, contributing to the pathogenesis of central sympathetic excitation [62]. Closely related, phosphorylated activation of epidermal growth factor receptor (EGFR) in the PVN of these rats further enhances RAS activity, aggravates neuroinflammation and ERS via downstream ERK1/2 signaling, ultimately mediating central sympathetic overexcitation [63]. Upstream of this regulatory axis, increased transforming growth factor-α (TGF-α) in the PVN of HF rats also induces EGFR phosphorylation, strengthens ERK1/2 activation, and upregulates proinflammatory cytokines and RAS components, leading to central sympathetic overactivation [64]. Besides these pathways, prostaglandin E2 in the PVN of chronic HF rats activates c-Jun N-terminal kinase (JNK) through the EP3 receptor (PTGER3), downregulates glutamic acid decarboxylase 1 (GAD1) and GABAA receptor α1 subunit (GABRA1) expression, and suppresses GABAergic inhibitory signaling, thereby increasing sympathetic nerve discharge and participating in central sympathetic overexcitation [65].

Regarding the crosstalk between transcription factors and receptors, elevated phosphorylation of inhibitor κB kinase β (IKKβ) in the PVN of rats with ischemic HF activates NF-κB p65. This induces neurotransmitter imbalance and oxidative stress by upregulating glutamate, norepinephrine, and tyrosine hydroxylase (TH) expression, promoting superoxide production, while reducing γ-aminobutyric acid (GABA) and its synthetic enzyme GAD67, thereby increasing renal sympathetic nerve activity [66]. In a similar model, AT1-R in the PVN of rats with myocardial infarction-induced HF interacts with NF-κB, further activating the NF-κB pathway by upregulating phosphorylated IKKβ (p-IKKβ). This synergistically promotes neurotransmitter imbalance and oxidative stress, mediating central sympathetic overexcitation [67]. In addition, upregulated hypoxia-inducible factor-1α (HIF-1α) in the PVN of rats with chronic HF following myocardial infarction binds to the promoter of N-methyl-D-aspartate receptor subtype 1 (NMDA-NR1) and enhances its expression. This strengthens glutamatergic signaling, increases basal sympathetic tone, and ultimately mediates central sympathetic overexcitation [68].

Current studies on central signaling pathways and transcription factors are mostly concentrated in the PVN, with relatively insufficient exploration of other key sympathetic regulatory nuclei such as the SFO, RVLM, and NTS. Meanwhile, the universality and consistency of existing findings across different heart failure models have not been systematically verified.

#### 3.2.4. Abnormal Post-Transcriptional and Post-Translational Modifications in the Central Nervous System: Sophisticated Reinforcement Mechanisms Underlying Sympathetic Pathological States

The development of central sympathetic excitation depends not only on changes at the gene transcription level but also on post-transcriptional and post-translational regulation, which plays a key role. By rapidly modulating target molecule function through non-coding RNA-mediated gene silencing, protein ubiquitination, and other pathways, these processes contribute to the pathological disruption of central sympathetic regulation following HF.

##### Abnormal Post-Transcriptional Gene Regulation Mediated by Non-Coding RNAs

In the PVN of CHF rats, an AngII/AT_1_R/HoxD10/miR-7b/GABBR1 regulatory axis exists. AngII upregulates homeobox protein D10 (HoxD10) and microRNA-7b (miR-7b) via AT1R. Post-transcriptionally, miR-7b targets the 3′UTR of GABAB receptor 1 (GABBR1) and represses its translation, resulting in reduced GABBR1 expression and weakened GABAergic inhibition, ultimately mediating central sympathetic excitation and cardiac dysfunction [69]. In addition, decreased expression of miR-133a in the PVN of CHF rats weakens its post-transcriptional inhibitory effect on the 3′UTR of angiotensinogen (AGT). This leads to upregulated AGT and AT1R expression, activates the local RAS, thereby increasing RSNA and mediating central sympathetic excitation [70].

##### Functional Imbalance of Protein Post-Translational Modifications

At the level of protein post-translational modifications, increased expression of sodium-glucose cotransporter 2 (SGLT2) in endothelial cells of the SFO in mice with hypertensive heart failure inhibits ubiquitin-dependent degradation of cGAS, aggravates neuroinflammation, and mediates central sympathetic excitation [71]. Meanwhile, in the PVN of CHF rats, AngII upregulates expression of protein inhibitor of nNOS (PIN), promotes depolymerization and ubiquitin-dependent degradation of nNOS, attenuates the sympathetic inhibitory effect mediated by nitric oxide (NO), and ultimately induces central sympathetic excitation [72].

These findings highlight the critical contributions of post-transcriptional and post-translational regulation to central sympathetic hyperactivity; however, most investigations focus on a small set of miRNAs and ubiquitination-related molecules in limited nuclei. The functional crosstalk between different epigenetic and protein-modifying pathways remains poorly defined, and their upstream regulatory networks and downstream pathological amplification effects require further mechanistic exploration.

### 3.3. Execution Stage: Neuronal Microenvironment Disorder and Sympathetic Output

#### 3.3.1. Imbalance of the Functional Microenvironment in Central Neurons: A Direct Manifestation of Sympathetic Neuronal Hyperexcitability

The excitatory homeostasis of central neurons relies on a microenvironment composed of neurotransmitters, receptors, ion channels, and glial cells. This homeostasis is disrupted in HF, characterized by weakened inhibitory transmitter effects, activation of excitatory systems, and abnormal receptor and ion channel functions. These changes lead to hyperexcitability of neurons in sympathetic regulatory centers and enhanced central sympathetic outflow. This section elaborates on the underlying pathological mechanisms from two aspects: the neurotransmitter and receptor system, and ion channel function.

##### Functional Imbalance of the Neurotransmitter and Receptor System

In rats with chronic heart failure, mRNA expression of the GABA(A) receptor α_1_ subunit and GABA(B1a)/GABA(B1b) receptor subtypes in the hypothalamic paraventricular nucleus (PVN) is downregulated. This weakens the tonic inhibition of sympathetic outflow mediated by GABA(A) and GABA(B) receptors, thereby mediating central sympathetic overactivation [73]. Meanwhile, in PVN neurons projecting to the rostral ventrolateral medulla (PVN-RVLM) of HF rats, astrocytic GABA uptake is enhanced and function of the GABAA receptor δ subunit is reduced. Together these cause a marked decrease in GABAA receptor-mediated tonic inhibitory currents, worsening GABAergic inhibition in the PVN and enhancing central sympathetic outflow [74]. In addition to impaired GABAergic inhibition, corticotropin-releasing hormone (CRH) in the PVN of rats with ischemic HF modulates neurotransmitter balance via type 1 receptors (CRH-R1). This elevates glutamate, norepinephrine, and tyrosine hydroxylase levels while reducing GABA and glutamic acid decarboxylase GAD67 levels, thereby activating renal sympathetic nerves, stimulating corticotropin release, and mediating central sympathetic excitation [75]. Furthermore, in salt-loaded Dahl salt-sensitive hypertensive HF rats, renal afferent nerves activate vasopressinergic neurons in the PVN. Excitation of the PVN-RVLM sympathetic pathway via vasopressin V1a/V1b receptors further induces central sympathetic excitation and contributes to HF progression [22].

##### Abnormal Regulation of Ion Channel Expression and Function

At the ion channel level, abnormal activation of N-methyl-D-aspartate receptor 1 (NMDAR1) in the PVN of HF rats, accompanied by upregulated TH and downregulated glutamic acid decarboxylase 67 (GAD67), significantly increases RSNA, elevates plasma NE and AngII levels, and mediates central sympathetic excitation and cardiac dysfunction [76]. Similarly, increased mRNA and protein expression of the NMDA receptor NR1 subunit in the PVN of HF rats further enhances glutamate-mediated sympathoexcitation, raises RSNA, and exacerbates central sympathetic overactivation [77]. Under CHF conditions, central AngII downregulates voltage-gated potassium channel Kv4.3 expression in the RVLM via the AT1R–ROS–p38 MAPK signaling pathway, reduces A-type potassium current, increases RVLM neuronal excitability, and ultimately mediates central sympathetic excitation [78].

Nevertheless, insights into neuronal microenvironmental dysfunction are predominantly derived from preclinical models, and clinical translatability to human heart failure remains undetermined. Furthermore, generalizability across distinct etiologies and clinical stages of heart failure has not been established.

### 3.4. Crosstalk and Networked Regulation Among Pathological Mechanisms

During central sympathetic overactivation in HF, pathological pathways do not act independently. Extensive crosstalk exists among ERS, oxidative stress, neuroinflammation, and MAPK signaling, which synergistically and sequentially drive abnormal increases in central sympathetic nerve activity. EV-mediated trans-organ signaling induces oxidative stress imbalance and neuroinflammation, while injured mitochondria trigger neuroinflammatory cascades by elevating ROS levels. ERS activates MAPK signaling to exacerbate neuroinflammation and central RAS imbalance, and directly induces neuronal hyperexcitability via a synergistic pathway involving upregulated oxidative stress, elevated inflammatory cytokines, and activated central RAS. Meanwhile, inflammatory cytokines and abnormal RAS activation reciprocally induce ERS, forming a pathological positive feedback loop. Crucially, RAS, oxidative stress, and MAPK signaling further disrupt neuronal microenvironmental homeostasis and cause ion channel dysfunction, thereby enhancing excitability of sympathetic regulatory neurons. In addition, MAPK signaling is jointly activated by ERS, oxidative stress, neuroinflammation, and RAS imbalance, acting as a core hub to integrate and transmit multiple pathological signals. Such multi-pathway crosstalk constitutes the networked pathological basis for central sympathetic overactivation in HF.

## 4. Targeted Intervention Strategies and Clinical Translation Potential for Central Sympathetic Hyperactivity in Heart Failure(HF)

**Figure 5:**
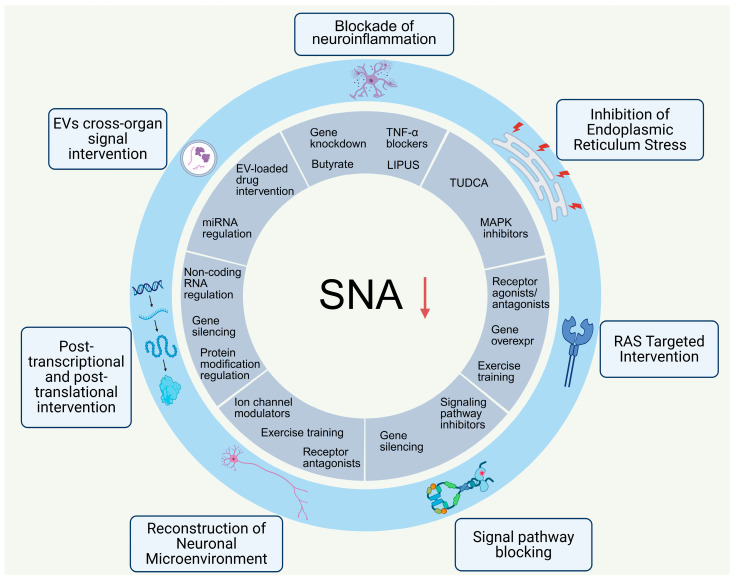
Multi-Dimensional Central Targeted Strategies for Mitigating Sympathetic Overactivity in Heart Failure. Schematic summarizing integrated therapeutic strategies targeting central sympathetic dysregulation. Note: ↓ indicates decreased activity. Abbreviations: SNA (sympathetic nerve activity), RAS (renin-angiotensin system), EVs (extracellular vesicles), TNF-α (tumor necrosis factor-α), LIPUS (low-intensity pulsed ultrasound), TUDCA (tauroursodeoxycholic acid), MAPK (mitogen-activated protein kinase), miRNA (microRNA), and overexpr (overexpression). Created in BioRender. Zhengwei Li, Renjun Wang. (2026) https://BioRender.com/gzdjor5 (accessed on 25 March 2026).

The central regulatory mechanisms underlying sympathetic overactivation in heart failure are multidimensional and network-based, with inflammation, abnormal signaling, and microenvironmental disturbances in key nuclei including SFO, PVN, RVLM, and NTS as the core pathological basis. Accordingly, central targeted interventions follow the core principles of reversing nuclear pathological abnormalities, blocking cascades of pathological pathways, and restoring sympathetic regulatory homeostasis. Based on seven major pathological mechanisms, diverse strategies have been developed, such as nucleus-targeted modulation, pathway blockade, molecular regulation, and nonpharmacological interventions, all of which have been verified in basic research to attenuate sympathetic hyperactivity and protect cardiac function (Figure 5). Although current strategies largely remain at the basic research stage, they provide abundant targets and research directions for precise neuromodulatory therapy in heart failure. Several nonpharmacological interventions and drug-repurposing strategies have already demonstrated preliminary prospects for clinical translation.

### 4.1. Targeted Intervention of Extracellular Vesicle-Mediated Trans-Organ Signaling

Focusing on EV-mediated trans-organ signaling linking peripheral cardiac injury to central sympathetic activation, interventions can effectively break the central-peripheral pathological vicious cycle by blocking aberrant EV signal delivery and reversing EV-induced central oxidative stress and inflammation. For the oxidative stress pathway, heart-derived EVs induce sympathetic excitation by delivering miRNAs that downregulate the Nrf2 pathway in the RVLM. Loading specific antagonists targeting miR-27a, miR-28a, and miR-34a (antagomir-27a, antagomir-28a, and antagomir-34a) into EVs can directly block the oxidative stress signal transmission mediated by EVs, thereby alleviating oxidative stress imbalance in the RVLM and reducing renal sympathetic nerve activity [38]. Furthermore, with regard to the inflammatory pathway, the aberrant microRNA profile of peripheral circulating exosomes in chronic heart failure mediates hyperinflammation in the RVLM. Inhibition of abnormally upregulated miR-214-3p using a miR-214-3p inhibitor, or restoration of downregulated let-7g-5p/let-7i-5p using let-7g-5p mimics and let-7i-5p mimics, can targetedly suppress the expression of proinflammatory genes (including Traf3, Smad2, and Mapk6) and alleviate central neuroinflammation in the RVLM, providing a novel direction for trans-organ precise interventions targeting exosomal miRNAs [40]. These miRNA-based interventions represent potentially clinically translatable emerging strategies that directly target the brain–heart axis. They may offer complementary value to current heart failure therapies by specifically addressing centrally driven sympathetic hyperactivity, which is often incompletely reversed by conventional peripheral pharmacotherapy alone.

### 4.2. Targeted Blockade of Central Neuroinflammation and Glial Activation

Given the initiating role of neuroinflammatory cascades mediated by glial activation in the SFO and PVN in sympathetic activation, interventions target core inflammatory molecules, receptors, and glial activation pathways to achieve blockade at the source of inflammation initiation and downstream inhibition of inflammatory effects. For upstream blockade, cGAS, PLA2G2A, and TNFR1 in the SFO represent highly promising central therapeutic targets, and knockdown of these molecules can directly block central inflammation triggered by peripheral injury signals, constituting a core potential strategy for ameliorating sympathetic overactivity [46,47,49]. Downstream inhibition focuses on regulating inflammatory amplification and sympathetic neuron activation in the PVN. Local application of TNF-α blockers in the PVN reverses inflammation-induced imbalance in receptors and neurotransmitters, directly inhibiting the activation of sympathetic premotor neurons to alleviate sympathetic activation [48]. Intestinal metabolite butyrate further targets PVN microglia, suppressing their inflammatory activation and downregulating overexpressed NMDAR1, thereby attenuating sympathetic excitation through dual anti-inflammatory and receptor-modulating effects [50]. Importantly, this pathway highlights the potential value of gut microbiota-targeted non-pharmacological interventions via the gut–brain axis for ameliorating central sympathetic dysfunction and improving heart failure prognosis. As a noninvasive physical intervention, low-intensity pulsed ultrasound (LIPUS) specifically inhibits PVN microglial activation and the P2x7/NLRP3 inflammatory pathway, exerting multiple effects including anti-inflammation, sympathetic inhibition, and antiarrhythmia [51]. It represents a non-pharmacological, rapidly translatable emerging central neuromodulatory strategy, with prominent clinical translational potential.

### 4.3. Inhibition of Central ERS and Blockade of Secondary Pathological Injury

Given the sustained positive feedback activation loop between central ERS and the MAPK-NF-κB pathway, targeted interventions can simultaneously ameliorate secondary pathological injuries induced by central inflammation and RAS imbalance through direct inhibition of ERS activation and blockade of abnormal downstream MAPK pathway activation. Central administration of the ERS inhibitor tauroursodeoxycholic acid (TUDCA) can target and suppress ERS activation in the SFO and PVN, thereby blocking abnormal MAPK pathway activation, reducing central inflammation and excessive RAS activation, and lowering plasma norepinephrine levels to alleviate sympathetic excitation [52]. Meanwhile, TUDCA can also inhibit ERS in the RVLM, reverse ERS-mediated inflammation and RAS activation in this region, improve cardiac autonomic nervous balance, correct arrhythmias and abnormal respiratory patterns, and alleviate myocardial hypertrophy and ventricular diastolic dysfunction. This provides a potential strategy for multi-target improvement of cardiopulmonary function abnormalities in heart failure [54]. In addition, specific inhibitors including PD98059, SB203580 and SP600125 selectively target and inhibit the key molecules of the MAPK pathway, namely p44/42 MAPK, p38 MAPK and JNK, respectively. This intervention can directly block ERS-mediated inflammatory and RAS activation signaling at the downstream level, and reversely alleviate ERS and neuroinflammation in the SFO and PVN, thereby reducing sympathetic overexcitation and improving cardiac function [53]. Collectively, these findings indicate that targeting central ERS-MAPK signaling represents a promising translatable therapeutic direction for correcting autonomic imbalance and treating advanced heart failure.

### 4.4. Nucleus-Specific Targeted Intervention for Central RAS Imbalance

In response to receptor imbalance and abnormal signaling activation of the central RAS across key nuclei, interventions focus on nucleus-specific regulation of RAS component expression, restoration of receptor balance, and blockade of aberrant signaling pathways to precisely restore sympathetic regulatory function in distinct nuclei. At the PVN level, targeting the Ang-(1-7)/Mas receptor pathway with the antagonist A-779 directly blocks its mediated sympathetic excitatory signals. Concurrent inhibition of the downstream cAMP-PKA pathway or NAD(P)H oxidase-derived superoxide anion production exerts synergistic effects [55]. As a gene intervention strategy with potential translational value, overexpression of ACE2 upregulates nNOS and enhances the NO-mediated sympathetic inhibitory pathway, reversing the impaired sympathetic suppression in the PVN and reducing renal sympathetic nerve activity [56]. At the SFO level, exercise training, as a classic nonpharmacological intervention, downregulates overexpressed AT1 receptors, inhibits excessive SFO neuronal activation, and reverses AngII-induced sympathetic excitation, making it suitable for long-term adjuvant intervention in heart failure [58]. Clinical studies further confirm that exercise training effectively reduces peripheral muscle sympathetic nerve activity (MSNA) in patients with heart failure [79], consistent with central sympathoinhibitory effects. At the RVLM level, activation of AT2R with selective agonist CGP42112 restores the AT1R/AT2R expression balance, exerting multiple effects including sympathetic inhibition, blood pressure reduction, and heart rate slowing [59]. At the NTS level, the use of clinically commonly used angiotensin II type 1 receptor-blocking drugs losartan or the specific antagonist CV11974 to block angiotensin II type 1 receptors can attenuate reflex regulation dysfunction mediated by the renin-angiotensin system, reduce basal sympathetic activity, inhibit reflex facilitation, and lower arterial blood pressure and renal sympathetic nerve activity [60,61]. In summary, these nucleus-specific RAS regulation strategies (including existing drug repurposing, emerging targeted formulations, and non-pharmacological interventions) provide diverse approaches and solid evidence for translating central autonomic balance mechanisms into clinical heart failure treatment regimens.

### 4.5. Cascade Blockade of Abnormal Central Core Signaling Pathways and Transcription Factors

Targeting aberrant activation of core signaling pathways including MAPK and NF-κB, as well as transcription factors such as HIF-1α in the PVN, strategies including pathway cascade blockade and targeted silencing of transcription factors can reverse pathological changes mediated by abnormal signaling, including neuroinflammation, ERS, and neurotransmitter imbalance, thereby restoring sympathetic regulatory function in the PVN. At the upstream blockade level, silencing ERK1/2 and its upstream epidermal growth factor receptor and transforming growth factor-α in the PVN blocks ERK1/2 signaling cascade activation at the source, simultaneously alleviating neuroinflammation, ERS, and abnormal RAS activation, and reducing plasma norepinephrine levels [62,63,64]. At the pathway inhibition level, blocking prostaglandin E2 receptor 3 or inhibiting c-Jun N-terminal kinase restores normal expression of GAD1 and GABRA1, reverses prostaglandin-mediated attenuation of GABAergic inhibitory signaling, and ameliorates sympathetic excitation [65]. Administration of NF-κB inhibitors (SN50, pyrrolidine dithiocarbamate) or AT1 receptor blockers (losartan) corrects NF-κB-mediated neurotransmitter imbalance, reduces oxidative stress, and directly decreases PVN-driven sympathetic nerve activity [66,67]. At the transcription factor targeting level, silencing HIF-1α in the PVN downregulates its target gene NMDA receptor expression, abolishes elevated baseline sympathetic tone and NMDA-induced sympathetic excitatory responses, providing a novel strategy for precise transcription factor-targeted intervention [68]. The above cascade blockade strategies targeting core signaling pathways and transcription factors in the PVN, including repurposed clinical drugs, pathway-specific inhibitors, and novel gene-targeted interventions, provide multifaceted and highly translatable intervention approaches for translating central signaling dysregulation into clinical heart failure therapies.

### 4.6. Precise Molecular Interventions for Abnormal Central Post-Transcriptional and Post-Translational Modifications

#### 4.6.1. Targeted Reversal of Non-Coding RNA-Mediated Post-Transcriptional Dysregulation

In response to miRNA-mediated post-transcriptional dysregulation in the PVN, strategies can be applied to target and reverse the gene-silencing effects of aberrant miRNAs and restore normal target gene expression, thereby achieving precise reversal of pathological regulatory axes—an important direction for molecular-level precision intervention. In heart failure, abnormal activation of the AngII/AT1R/HoxD10/miR-7b pathway in the PVN suppresses GABBR1 expression. Infusion of miR-7b antagomir into the PVN, blockade of AT1R, or silencing of HoxD10 can intervene in this regulatory axis at different nodes, restore normal GABBR1 levels, and reverse weakened GABAergic inhibition [69]. Furthermore, overexpression of miR-133a in the PVN restores its post-transcriptional inhibitory effect on angiotensinogen and AT1R, thereby inhibiting abnormal local RAS activation in the PVN and reducing renal sympathetic nerve activity [70].

#### 4.6.2. Repair and Regulation of Functional Imbalance in Protein Post-Translational Modifications

Based on impaired molecular function mediated by abnormal post-translational modifications such as protein ubiquitination and dimerization, interventions mainly aim to restore normal post-translational modifications and reverse target protein dysfunction. Meanwhile, some strategies repurpose existing clinical drugs to improve clinical translation efficiency. Inhibition of SGLT2 in the SFO (e.g., with the clinically used drug empagliflozin) restores ubiquitin-mediated degradation of cGAS, alleviates neuroinflammation and oxidative stress induced by SGLT2 overexpression, thereby reducing sympathetic excitation and improving cardiac function, providing new evidence for central targeting of SGLT2 inhibitors [71]. In addition, central nNOS expression and function are weakened in heart failure due to abnormal post-translational modifications. Stabilizing nNOS dimers and targetedly regulating its post-translational modification process to restore nNOS function can re-establish the NO-mediated sympathetic inhibitory pathway, representing a potential approach for ameliorating sympathetic hyperactivity [72].

### 4.7. Reconstruction Strategy of Central Neuronal Functional Microenvironment Homeostasis

#### 4.7.1. Repair and Regulation of Neurotransmitter and Receptor System Function

Based on the core pathological features of weakened inhibitory neurotransmitter function and imbalanced excitatory neurotransmission within the PVN, interventions focus on restoring inhibitory transmitter function and correcting multi-transmitter imbalance to reestablish sympathetic regulatory homeostasis in the neuronal microenvironment. In heart failure, sympathetic inhibition mediated by GABA(A) and GABA(B) receptors in the PVN is significantly attenuated. Targeted regulation to restore or enhance GABAergic inhibitory function represents an important potential strategy for ameliorating sympathetic hyperactivity [73]. Blockade of GABA transporter 3 (GAT-3) inhibits abnormally enhanced GABA uptake by astrocytes, restores tonic inhibition via GABA(A) receptors, and thereby reduces sympathetic outflow from the PVN [74]. Furthermore, blockade of corticotropin-releasing hormone receptor 1 (CRH-R1) in the PVN corrects upstream imbalance among multiple neurotransmitters including glutamate, norepinephrine, and GABA, directly reducing renal sympathetic nerve activity [75]. Blockade of vasopressin V1a and V1b receptors inhibits activation of the PVN-RVLM sympathetic pathway mediated by renal afferent nerves, providing exploitable targets for intervention in reno-cerebral axis-driven sympathetic hyperactivity [22].

#### 4.7.2. Targeted Correction and Physiological Regulation of Ion Channel Dysfunction

In response to neuronal hyperexcitability mediated by abnormal ion channel expression and function in the PVN and RVLM, core intervention strategies include blocking excitatory ion channels, restoring normal potassium channel function, and combining nonpharmacological approaches to achieve physiological regulation of ion channel activity. Administration of the NMDA receptor antagonist AP5 within the PVN downregulates aberrantly activated NMDA receptors, modulates related protein expression, reduces renal sympathetic nerve activity, and improves cardiac function, representing a classic potential ion channel-targeted intervention [76]. Exercise training normalizes overexpressed NMDA receptors in the PVN and attenuates glutamate-mediated sympathetic excitation, serving as a noninvasive and widely applicable nonpharmacological intervention [77]. In HF, Kv4.3 channels in the RVLM are abnormally downregulated via the AT1R-ROS-p38 MAPK pathway. Targeted blockade of this pathway reverses Kv4.3 channel expression and dysfunction, restores potassium current homeostasis in RVLM neurons, and inhibits neuronal hyperexcitability, providing a novel precise pathway-targeted strategy for ameliorating sympathetic hyperactivity [78].

### 4.8. Translational Hurdles and Limitations of Central Targeted Therapies

Despite the promising preclinical efficacy of central neuromodulatory strategies including SGLT2 inhibitors, miRNA antagomirs, and TUDCA, substantial translational barriers persist that limit their clinical implementation. Chronic suppression of central inflammatory or signaling pathways carries inherent risks of off-target effects and systemic side effects, as core regulatory molecules are broadly expressed across multiple brain regions and peripheral tissues, potentially disrupting physiological autonomic homeostasis and precipitating unintended cardiovascular or neurological complications. Beyond safety considerations, pharmacoeconomic challenges further hinder the clinical adoption of advanced biologics such as miRNA therapeutics and CNS-targeted delivery systems, which are associated with high manufacturing costs, complex formulation demands, and uncertain long-term safety profiles compared with repurposed small-molecule drugs. Compounding these limitations, insufficient blood–brain barrier penetrability and suboptimal nucleus-specific targeting further compromise the therapeutic index and translational potential of current central intervention approaches.

## 5. Research Prospects and Challenges

Although multidimensional and network-based pathological mechanisms underlying central sympathetic activation in heart failure have been elucidated, critical scientific challenges remain in the refined analysis of core mechanisms and the clinical translation of intervention strategies. The integration of interdisciplinary technologies and innovative exploration of specific regulatory axes provide new directions for this field. In mechanism elucidation, the functional heterogeneity of cell subtypes in key nuclei such as the PVN and SFO remains unclear, and the spatiotemporal regulatory sequence and synergistic mode of multiple mechanisms (e.g., neuroinflammation, RAS imbalance) need to be clarified. Cutting-edge technologies such as single-cell RNA sequencing should be employed to dissect the hierarchical regulatory relationships among nuclei, cell subtypes, and molecular signals [80]. In clinical translation, poor blood–brain barrier penetration and insufficient specificity of central targets are major technical bottlenecks. There is an urgent need to develop nucleus/cell subtype-targeted delivery systems and innovate precise neuromodulation technologies such as optogenetics—the sympathetic inhibitory efficacy of neural stimulation has been experimentally verified [81]. Meanwhile, it is necessary to screen and identify cerebrospinal fluid molecular biomarkers to build a translational bridge between basic research and clinical practice. Furthermore, issues such as the crosstalk of multiple mechanisms, regulatory heterogeneity among heart failure subtypes, and key nodes of trans-organ signal transduction remain unclear, resulting in limited clinical benefits of single-target interventions. Interdisciplinary technologies support mechanism analysis and the development of new strategies. The gut microbiota-brain-heart axis has shown promising intervention potential [50], providing an important frontier direction for precise neuromodulation research in heart failure.

## 6. Conclusions

Sympathetic overactivation constitutes a major factor involved in the onset and progression of heart failure. Structural remodeling and functional abnormalities in the central regulatory network act to trigger and maintain this pathological process. The sympathetic regulatory neural circuit composed of core nuclei including the SFO, PVN, RVLM, and NTS forms an interactive, positively regulated networked pathological system through seven major mechanisms—neuroinflammatory activation, endoplasmic reticulum stress, central RAS imbalance, and others. This network plays an instrumental role in promoting central sympathetic overactivation and establishes a brain–heart axis vicious cycle with peripheral cardiac injury, exacerbating the progression of heart failure. Central targeted intervention strategies developed based on these mechanisms include nucleus-specific pathway blockade, molecular targeted regulation, noninvasive physical intervention, and drug repurposing. Basic experiments have verified that these approaches can reverse pathological abnormalities in central nuclei and restore sympathetic regulatory homeostasis. Low-intensity pulsed ultrasound, exercise training, SGLT2 inhibitors, and others have demonstrated favorable clinical translation potential, providing abundant targets and practical strategies for precise neuromodulatory therapy in heart failure. In summary, central sympathetic overactivation in heart failure is characterized by multidimensionality, networking, and spatiotemporal specificity. Related studies have deeply revealed the pathological remodeling nature of the heart failure brain–heart axis, laying a theoretical foundation for the development of novel precise neuromodulatory therapies. With further elucidation of central regulatory mechanisms and the development of interdisciplinary translational technologies, interventions targeting the central sympathetic regulatory network are expected to break the brain–heart axis pathological vicious cycle, offering new breakthrough directions for precise clinical treatment and prognosis improvement in heart failure.

## Figures and Tables

**Table 1:** Central renin–angiotensin system (RAS) imbalance mediating central sympathetic overactivation in heart failure.

Disease Model	Key Brain Region	Core Molecular Changes	Signaling Pathways/Mechanisms	Functional Outcome	References
CHF rat	PVN	Ang-(1-7) ↑; Mas receptor ↑	cAMP-PKA; NAD(P)H oxidase-derived superoxide	Enhanced cardiac sympathetic afferent reflex; increased sympathetic activation	[55]
CHF rat	PVN	ACE2 ↓; Ang-(1-7) receptor ↓; nNOS ↓	NO-mediated sympathetic inhibitory pathway impaired	Amplified sympathetic excitation	[56]
HF rat	PVN	NF-κB activation; AT1R ↑; ROS ↑	RAS–inflammatory cytokine crosstalk; positive feedback loop	Neurohumoral excitation; increased central sympathetic activation	[57]
CHF rat	SFO	AT1R ↑	Enhanced tonic activation of Ang II	SFO neuronal activation; increased RSNA and blood pressure	[58]
CHF rat	RVLM	AT1R ↑; AT2R ↓	Loss of AT2R-mediated sympathetic inhibition via arachidonic acid pathway	Dual mechanism leading to sympathetic overactivation	[59]
CHF rat	NTS	AT1R ↑	Enhanced interaction between CSAR and chemoreflex	Amplified sympathetic excitatory effect	[60]
HF rat	NTS	ACE ↑, local RAS activation	Endogenous Ang II activates AT1R signaling	Increased RSNA and arterial blood pressure	[61]

Note:↑ indicates upregulation, activation, or increased effect; ↓ indicates downregulation, inactivation, or impaired function. Abbreviations: CHF (chronic heart failure); HF (heart failure); PVN (paraventricular nucleus); SFO (subfornical organ); RVLM (rostral ventrolateral medulla); NTS (nucleus tractus solitarius); Ang-(1-7) (angiotensin-(1-7)); ACE2 (angiotensin-converting enzyme 2); nNOS (neuronal nitric oxide synthase); NF-κB (nuclear factor-κB); AT1R (angiotensin II type 1 receptor); AT2R (angiotensin II type 2 receptor); ROS (reactive oxygen species); cAMP-PKA (cyclic adenosine monophosphate-protein kinase A); NAD(P)H (nicotinamide adenine dinucleotide phosphate); CSAR (cardiac sympathetic afferent reflex); RSNA (renal sympathetic nerve activity); RAS (renin–angiotensin system).

**Table 2:** Abnormal activation of central signaling pathways and transcription factors in mediating sympathetic overactivation in heart failure.

Disease Model	Key Brain Region	Core Molecular Changes	Signaling Pathways/Mechanisms	Functional Outcome	References
Myocardial infarction-induced HF rat	PVN	ERK1/2 MAPK ↑	ERK1/2 pathway mediates upregulation of RAS components and inflammatory mediators	Increased PVN neuronal excitability; elevated plasma norepinephrine; enhanced sympathetic drive	[62]
Myocardial infarction-induced HF rat	PVN	EGFR ↑	EGFR activation promotes downstream ERK1/2 signaling; enhances RAS activity; aggravates neuroinflammation and ERS	Central sympathetic overactivation	[63]
Myocardial infarction-induced HF rat	PVN	TGF-α ↑	TGF-α upregulation induces EGFR phosphorylation and ERK1/2 activation; upregulates proinflammatory cytokines and RAS components	Central sympathetic overactivation	[64]
CHF rat	PVN	JNK ↑	PGE2 acts through EP3 (PTGER3) to activate JNK; downregulates GAD1 and GABRA1; suppresses GABAergic inhibitory tone	Increased sympathetic nerve discharge; central sympathetic overexcitation	[65]
Ischemic HF rat	PVN	p-IKKβ ↑; NF-κB p65 ↑	IKKβ/NF-κB pathway mediates neurotransmitter imbalance and oxidative stress	Increased renal sympathetic nerve activity	[66]
Myocardial infarction-induced HF rat	PVN	AT1R ↑; p-IKKβ ↑; NF-κB ↑	AT1-R/IKKβ/NF-κB crosstalk promotes neurotransmitter imbalance and oxidative stress	Central sympathetic overexcitation	[67]
Myocardial infarction-induced CHF rat	PVN	HIF-1α ↑; NMDA-NR1 ↑	HIF-1α promotes NMDA-NR1 transcription; enhances glutamatergic signaling	Elevated basal sympathetic tone; central sympathetic overexcitation	[68]

Note: ↑ indicates upregulation, phosphorylation, or hyperactivation. Abbreviations: HF (heart failure); CHF (chronic heart failure); PVN (paraventricular nucleus); ERK1/2 MAPK (extracellular regulated protein kinases 1/2 mitogen-activated protein kinase); RAS (renin–angiotensin system); EGFR (epidermal growth factor receptor); ERS (endoplasmic reticulum stress); TGF-α (transforming growth factor-α); PGE2 (prostaglandin E2); EP3 (PTGER3) (prostaglandin E2 receptor 3); JNK (c-Jun N-terminal kinase); GAD1 (glutamate decarboxylase 1); GABRA1 (GABA A receptor α1 subunit); GABA (γ-aminobutyric acid); IKKβ (inhibitor of nuclear factor-κB kinase subunit β); NF-κB (nuclear factor-κB); AT1R (angiotensin II type 1 receptor); HIF-1α (hypoxia-inducible factor-1α); NMDA-NR1 (N-methyl-D-aspartate receptor 1).

## Data Availability

No new data were created or analyzed in this study. Data sharing is not applicable to this article.

## Abbreviations

The following abbreviations are used in this manuscript:

ACE	Angiotensin-converting enzyme
ACE2	Angiotensin-converting enzyme 2
AGT	Angiotensinogen
Ang II	Angiotensin II
Ang-(1-7)	Angiotensin-(1-7)
AP	Area postrema
AT1R	Angiotensin II type 1 receptor
AT2R	Angiotensin II type 2 receptor
cAMP	Cyclic adenosine monophosphate
cGAS	Cyclic GMP-AMP synthase
CHF	Chronic heart failure
CNS	Central nervous system
COX-2	Cyclooxygenase-2
CRH	Corticotropin-releasing hormone
CRH-R1	Corticotropin-releasing hormone receptor 1
CSAR	Cardiac sympathetic afferent reflex
CVLM	Caudal ventrolateral medulla
DHODH	Dihydroorotate dehydrogenase
dsDNA	Double-stranded DNA
EGFR	Epidermal growth factor receptor
ERS	Endoplasmic reticulum stress
EVs	Extracellular vesicles
GABA	γ-aminobutyric acid
GABRA1	GABA A receptor α1 subunit
GABBR1	GABAB receptor 1
GAD1	Glutamate decarboxylase 1
GAD67	67 kDa glutamate decarboxylase
GAT-3	GABA transporter 3
gp91phox	NADPH oxidase subunit gp91phox
GRP78	Glucose-regulated protein 78
HF	Heart failure
HIF-1α	Hypoxia-inducible factor-1α
HoxD10	Homeobox protein D10
IKKβ	Inhibitor of nuclear factor-κB kinase subunit β
IL-1β	Interleukin-1beta
IL-6	Interleukin-6
IML	Intermediolateral column of the spinal cord
JNK	c-Jun N-terminal kinase
LIPUS	Low-intensity pulsed ultrasound
LSG	Left stellate ganglion
MAPK	Mitogen-activated protein kinase
MasR	Mas receptor
miR	MicroRNA
miR-7b	MicroRNA-7b
miR-133a	MicroRNA-133a
miR-214-3p	MicroRNA-214-3p
miR-27a	MicroRNA-27a
miR-28a	MicroRNA-28a
miR-34a	MicroRNA-34a
mtDNA	Mitochondrial DNA
NAD(P)H	Nicotinamide adenine dinucleotide phosphate
NF-κB	Nuclear factor-κB
NMDAR1	N-methyl-D-aspartate receptor 1
NMDA-NR1	N-methyl-D-aspartate receptor subtype 1
NO	Nitric oxide
nNOS	Neuronal nitric oxide synthase
NQO1	NAD(P)H quinone dehydrogenase 1
Nrf2	Nuclear factor erythroid 2-related factor 2
NTS	Nucleus tractus solitarius
p-IKKβ	Phosphorylated inhibitor κB kinase β
PKA	Protein kinase A
PLA2G2A	Secreted phospholipase A2 group IIA
PTGER3	Prostaglandin E2 receptor 3 (EP3)
PVN	Paraventricular nucleus of the hypothalamus
RAAS	Renin–angiotensin–aldosterone system
RAS	Renin–angiotensin system
ROS	Reactive oxygen species
RSNA	Renal sympathetic nerve activity
RVLM	Rostral ventrolateral medulla
SFO	Subfornical organ
SGLT2	Sodium-glucose cotransporter 2
SNA	Sympathetic nerve activity
SNS	Sympathetic nervous system
Smad2	SMAD family member 2
TGF-α	Transforming growth factor-α
TH	Tyrosine hydroxylase
TNF-α	Tumor necrosis factor-α
TNFR1	Tumor necrosis factor receptor 1
Traf3	TNF receptor-associated factor 3
TUDCA	Tauroursodeoxycholic acid
XBP-1s	Spliced X-box binding protein 1
